# 6-Bromo­pyridine-2-carbaldehyde phenyl­hydrazone

**DOI:** 10.1107/S1600536812026517

**Published:** 2012-06-20

**Authors:** Rodolfo Moreno-Fuquen, Manuel N. Chaur, Elkin L. Romero, Fabio Zuluaga, Javier Ellena

**Affiliations:** a Departamento de Química, Facultad de Ciencias, Universidad del Valle, Apartado 25360, Santiago de Cali, Colombia; b Instituto de Física de São Carlos, IFSC, Universidade de São Paulo, USP, São Carlos, SP, Brazil

## Abstract

The title compound, C_12_H_10_BrN_3_, is essentially planar (r.m.s. deviation of all non-H atoms = 0.0174 Å), with a dihedral angle of 0.5 (2)° between the two aromatic rings. In the crystal, mol­ecules are linked by weak N—H⋯N inter­actions, forming a zigzag chain running parallel to [001].

## Related literature

For bond-length data, see: Allen *et al.* (1987 ▶). For related structures, see: Yu *et al.* (2005 ▶); Fun *et al.* (2012 ▶). For the design of mol­ecular dynamic systems, see: Hirose (2010 ▶). For the principles of synthetic mol­ecular structures with dynamic properties, see: Kay *et al.* (2007 ▶). For configurational changes by UV light and heat, see: Chaur *et al.* (2011 ▶); Lehn (2006 ▶); Dugave & Demange (2003 ▶). For graph-set notation, see: Etter (1990 ▶). 
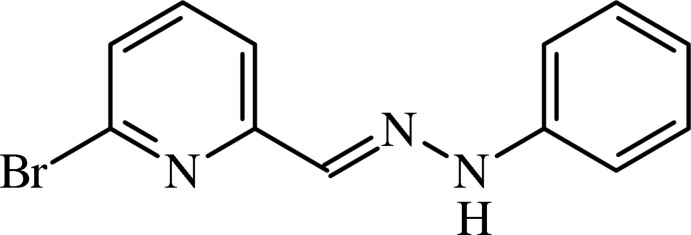


## Experimental

### 

#### Crystal data

C_12_H_10_BrN_3_
*M* *_r_* = 276.13
Orthorhombic, 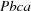

*a* = 14.6418 (3) Å
*b* = 7.8407 (1) Å
*c* = 20.0645 (4) Å
*V* = 2303.44 (7) Å^3^
*Z* = 8
Mo *K*α radiation
μ = 3.54 mm^−1^
*T* = 295 K
0.33 × 0.30 × 0.23 mm

#### Data collection

Nonius KappaCCD diffractometer
Absorption correction: multi-scan (*SADABS*; Sheldrick, 1996 ▶) *T*_min_ = 0.382, *T*_max_ = 0.544
28166 measured reflections
2339 independent reflections
1903 reflections with *I* > 2σ(*I*)
*R*_int_ = 0.048

#### Refinement

*R*[*F* ^2^ > 2σ(*F* ^2^)] = 0.039
*wR*(*F* ^2^) = 0.110
*S* = 1.03
2339 reflections
145 parameters
H-atom parameters constrained
Δρ_max_ = 0.44 e Å^−3^
Δρ_min_ = −0.72 e Å^−3^

### 

Data collection: *COLLECT* (Nonius, 2000 ▶); cell refinement: *SCALEPACK* (Otwinowski & Minor, 1997 ▶); data reduction: *DENZO* (Otwinowski & Minor, 1997 ▶) and *SCALEPACK*; program(s) used to solve structure: *SHELXS97* (Sheldrick, 2008 ▶); program(s) used to refine structure: *SHELXL97* (Sheldrick, 2008 ▶); molecular graphics: *ORTEP-3 for Windows* (Farrugia, 1997 ▶) and *Mercury* (Macrae *et al.*, 2006 ▶); software used to prepare material for publication: *WinGX* (Farrugia, 1999 ▶).

## Supplementary Material

Crystal structure: contains datablock(s) I, global. DOI: 10.1107/S1600536812026517/gg2083sup1.cif

Structure factors: contains datablock(s) I. DOI: 10.1107/S1600536812026517/gg2083Isup2.hkl

Supplementary material file. DOI: 10.1107/S1600536812026517/gg2083Isup3.cml

Additional supplementary materials:  crystallographic information; 3D view; checkCIF report

## Figures and Tables

**Table 1:** Hydrogen-bond geometry (Å, °)

*D*—H⋯*A*	*D*—H	H⋯*A*	*D*⋯*A*	*D*—H⋯*A*
N1—H1⋯N3^i^	0.86	2.34	3.180 (3)	166

Symmetry code: (i) 
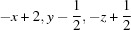
.

**Fig. 1.**
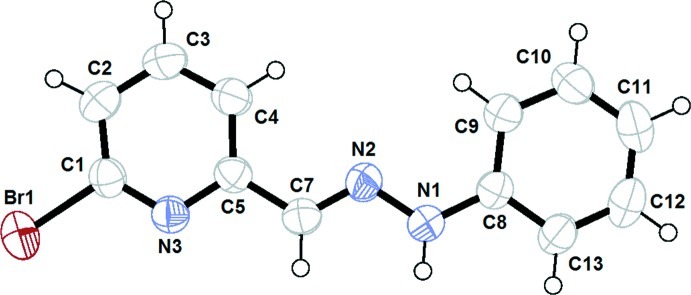
Molecular conformation and atom numbering scheme for (I) with displacement ellipsoids drawn at the 50% probability level. H atoms are shown as spheres of arbitrary radius.

**Fig. 2.**
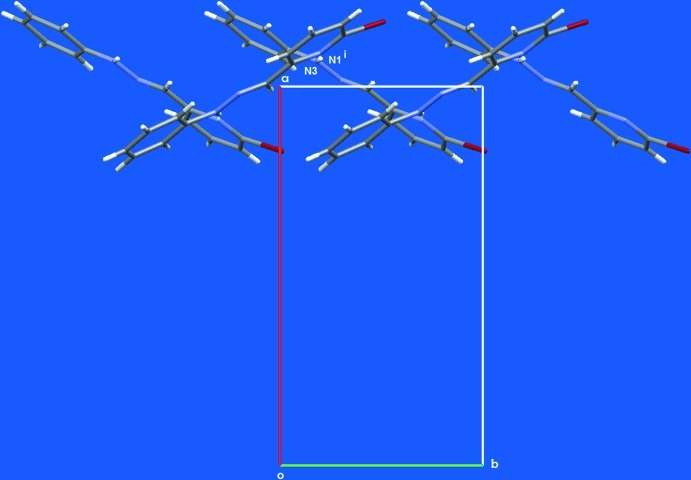
Part of the crystal structure of (I), showing the formation of one-dimensional zigzag chain running parallel to (001). Symmetry code: (i) -x+2,+y-1/2,-z+1/2.

**Fig. 3.**
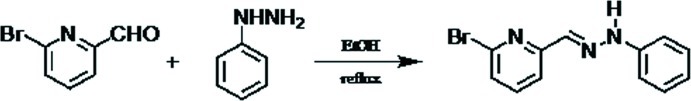
Synthesis of the 6-bromo-pyridinecarbaldehyde phenylhydrazone

## supplementary crystallographic information

### Comment

The design of molecular dynamic systems that operate by external stimuli without producing chemical waste is one of the questions of great interest in the nanotechnology field (Hirose, 2010), since these molecular systems are molecules that can exhibit structural (configurational) and chemical (constitutional) changes by the modification of external factors (presence of other molecules or metal ions, heat and/or light). Among the compounds that have attracted interest in this regard are the hydrazones which contain the imine group (-C=N-) (Kay *et al.*, 2007). This group can undergo reversible configurational changes induced by UV light and heat (Chaur *et al.*, 2011). For hydrazones prepared from pyridinecarboxaldehydes and pyridine or phenyl hydrazines the E/Z photoisomerization is favored by an intermolecular hydrogen bond between the hydrazine and the nitrogen of the pyridinecarboxaldehyde group resulting in a metastable state which can be returned to its original state by heating in solution (Chaur *et al.*, 2011; Lehn, 2006; Dugave & Demange, 2003). Besides, these compounds can exhibit dynamic properties since they can undergo coordination with suitable metals and the reversibility of the imine group gives them other features that can be used in the development of molecular machines or in the storage of information (Chaur *et al.*, 2011). The compound reported in this paper is part of a series of compounds that are currently being prepared in our group and that exhibit dynamic properties such as photoisomerization, constitutional changes and metal coordination in order to build supramolecular systems of multiple dynamics. Herein we report the synthesis and crystal structure of 6-bromo-pyridinecarbaldehyde phenylhydrazone (I), Fig 1. In the molecular structure of (I), the bromopyridyl ring is planar (r.m.s. deviation of all non-hydrogen atoms = 0.0020 Å) like the central bridge (C5/C7/N2/N1/C8) (r.m.s. deviation of all non-hydrogen atoms = 0.005 Å) with a dihedral angle of 0.6 (2)° between these two planes. The central bridge forms a dihedral angle of 0.8 (2)° with the benzene ring and the bromopyridyl and benzene rings form a dihedral angle of 0.5 (2)°. The bond lengths agree with the literature values (Allen *et al.*, 1987) and are comparable with the related structures (Yu *et al.*, 2005; Fun *et al.*, 2012). In the crystal packing (Fig. 2), the molecules are linked by weak N—H···N interactions (Table 1). Indeed, in this substructure, atom N1 in the molecule at (x,y,z) links to N3 atom in the molecule at (-x+2,+y-1/2,-z+1/2). The propagation of this interaction forms C(6) (Etter, 1990), continuous one-dimensional zigzag chain running parallel to [001].

### Experimental

The hydrazone under study was prepared by the condensation reaction of 6-bromo-2-pyridinecarboxaldehyde with phenylhydrazine in ethanol according to Fig. 3, obtaining a yellow solid with a yield of 81%. ^1^H NMR (400 MHz, DMSO-d_6_) δppm: 10.83 (s, 1H, NH), 7.92 (d, J= 8.03 Hz, 1H), 7.78 (s, 1H), 7.71 (t, J= 7.78 Hz, 1H), 7.47 (d, J= 7.78 Hz, 1H), 7.26 (m, 2H), 7.13 (d, J= 8.28 Hz, 2H), 6.83 (t, J= 7.28 Hz, 1H). NMR ^13^C (100 MHz, DMSO-d_6_) δppm: 156.16, 144.31, 140.77, 139.60, 134.52, 129.27, 126.07, 119.99, 117.80, 112.52. IR (KBr) N(cm^-1^): 3228 (N-H), 3038 and 2971 (=C-H), 1558 y 1601 (C=C y C=N). Melting point: 462 (1) K. Elemental analysis: Calculated: C 52.20 %, H 3.65 %, N 15.22 %; found: C 51.98 %, H 3.46 %, N 15.07 %.

### Refinement

All H-atoms were positioned geometrically using riding model with [C—H = 0.93 Å with *U*_iso_(H) = 1.2*U*_eq_(C)].

### Crystal data

**Table:**

C_12_H_10_BrN_3_	*D*_x_ = 1.593 Mg m^−^^3^
*M**_r_* = 276.13	Melting point: 497(1) K
Orthorhombic, *P**b**c**a*	Mo *K*α radiation, λ = 0.71073 Å
Hall symbol: -P 2ac 2ab	Cell parameters from 9233 reflections
*a* = 14.6418 (3) Å	θ = 3.6–26.4°
*b* = 7.8407 (1) Å	µ = 3.54 mm^−^^1^
*c* = 20.0645 (4) Å	*T* = 295 K
*V* = 2303.44 (7) Å^3^	Block, black
*Z* = 8	0.33 × 0.30 × 0.23 mm
*F*(000) = 1104	

### Data collection

**Table:**

Nonius KappaCCD diffractometer	2339 independent reflections
Radiation source: fine-focus sealed tube	1903 reflections with *I* > 2σ(*I*)
Graphite monochromator	*R*_int_ = 0.048
CCD rotation images, thick slices scans	θ_max_ = 26.4°, θ_min_ = 3.6°
Absorption correction: multi-scan (*SADABS*; Sheldrick, 1996)	*h* = −18→17
*T*_min_ = 0.382, *T*_max_ = 0.544	*k* = −9→9
28166 measured reflections	*l* = −24→25

### Refinement

**Table:**

Refinement on *F*^2^	Primary atom site location: structure-invariant direct methods
Least-squares matrix: full	Secondary atom site location: difference Fourier map
*R*[*F*^2^ > 2σ(*F*^2^)] = 0.039	Hydrogen site location: inferred from neighbouring sites
*wR*(*F*^2^) = 0.110	H-atom parameters constrained
*S* = 1.03	*w* = 1/[σ^2^(*F*_o_^2^) + (0.052*P*)^2^ + 1.6466*P*] where *P* = (*F*_o_^2^ + 2*F*_c_^2^)/3
2339 reflections	(Δ/σ)_max_ < 0.001
145 parameters	Δρ_max_ = 0.44 e Å^−^^3^
0 restraints	Δρ_min_ = −0.72 e Å^−^^3^

### Special details

**Table:**

Geometry. All esds (except the esd in the dihedral angle between two l.s. planes) are estimated using the full covariance matrix. The cell esds are taken into account individually in the estimation of esds in distances, angles and torsion angles; correlations between esds in cell parameters are only used when they are defined by crystal symmetry. An approximate (isotropic) treatment of cell esds is used for estimating esds involving l.s. planes.
Refinement. Refinement of *F*^2^ against ALL reflections. The weighted *R*-factor *wR* and goodness of fit *S* are based on *F*^2^, conventional *R*-factors *R* are based on *F*, with *F* set to zero for negative *F*^2^. The threshold expression of *F*^2^ > σ(*F*^2^) is used only for calculating *R*-factors(gt) *etc*. and is not relevant to the choice of reflections for refinement. *R*-factors based on *F*^2^ are statistically about twice as large as those based on *F*, and *R*- factors based on ALL data will be even larger.

### Fractional atomic coordinates and isotropic or equivalent isotropic displacement parameters (Å^2^)

**Table:**

	*x*	*y*	*z*	*U*_iso_*/*U*_eq_	
Br1	0.82948 (2)	1.00944 (4)	0.314727 (18)	0.07300 (18)	
N1	1.06677 (16)	0.1846 (3)	0.32501 (10)	0.0535 (5)	
H1	1.0780	0.2044	0.2837	0.064*	
N2	1.01869 (15)	0.2984 (3)	0.36079 (10)	0.0506 (5)	
N3	0.91170 (14)	0.6977 (3)	0.33261 (10)	0.0468 (5)	
C1	0.86323 (18)	0.8132 (3)	0.36454 (13)	0.0496 (6)	
C2	0.8363 (2)	0.8049 (4)	0.43023 (14)	0.0620 (7)	
H2	0.8017	0.8907	0.4499	0.074*	
C3	0.8635 (2)	0.6620 (4)	0.46526 (14)	0.0674 (8)	
H3	0.8471	0.6495	0.5098	0.081*	
C4	0.9144 (2)	0.5390 (4)	0.43455 (13)	0.0577 (7)	
H4	0.9332	0.4428	0.4579	0.069*	
C5	0.93799 (17)	0.5597 (3)	0.36750 (12)	0.0467 (5)	
C7	0.99160 (18)	0.4341 (4)	0.33110 (12)	0.0506 (6)	
H7	1.0061	0.4522	0.2865	0.061*	
C8	1.09823 (17)	0.0360 (3)	0.35418 (12)	0.0460 (5)	
C9	1.0819 (2)	−0.0034 (3)	0.42067 (14)	0.0568 (7)	
H9	1.0468	0.0692	0.4469	0.068*	
C10	1.1179 (2)	−0.1504 (4)	0.44746 (15)	0.0696 (8)	
H10	1.1073	−0.1750	0.4921	0.084*	
C11	1.1688 (2)	−0.2614 (4)	0.41011 (18)	0.0704 (8)	
H11	1.1925	−0.3603	0.4289	0.084*	
C12	1.1841 (2)	−0.2232 (4)	0.34413 (17)	0.0670 (8)	
H12	1.2184	−0.2976	0.3181	0.080*	
C13	1.14946 (19)	−0.0769 (4)	0.31608 (13)	0.0553 (7)	
H13	1.1604	−0.0533	0.2714	0.066*	

### Atomic displacement parameters (Å^2^)

**Table:**

	*U*^11^	*U*^22^	*U*^33^	*U*^12^	*U*^13^	*U*^23^
Br1	0.0783 (3)	0.0622 (2)	0.0785 (3)	0.01901 (15)	−0.00590 (15)	0.00948 (14)
N1	0.0623 (14)	0.0528 (13)	0.0454 (11)	0.0095 (11)	0.0048 (9)	−0.0002 (10)
N2	0.0556 (12)	0.0466 (12)	0.0495 (11)	0.0035 (10)	−0.0006 (10)	−0.0039 (10)
N3	0.0477 (11)	0.0483 (12)	0.0444 (10)	−0.0002 (9)	−0.0039 (9)	−0.0015 (9)
C1	0.0487 (13)	0.0479 (14)	0.0521 (14)	0.0009 (11)	−0.0069 (11)	−0.0008 (11)
C2	0.0704 (19)	0.0600 (17)	0.0557 (16)	0.0063 (14)	0.0061 (13)	−0.0107 (13)
C3	0.088 (2)	0.0676 (19)	0.0460 (14)	0.0066 (17)	0.0106 (14)	−0.0021 (14)
C4	0.0731 (18)	0.0524 (14)	0.0475 (14)	0.0013 (14)	0.0007 (13)	0.0024 (12)
C5	0.0491 (13)	0.0472 (13)	0.0440 (13)	−0.0023 (11)	−0.0036 (10)	−0.0007 (11)
C7	0.0548 (15)	0.0530 (14)	0.0439 (12)	0.0004 (12)	−0.0016 (11)	−0.0019 (12)
C8	0.0439 (13)	0.0461 (13)	0.0479 (13)	−0.0012 (10)	−0.0028 (10)	−0.0045 (11)
C9	0.0661 (17)	0.0505 (16)	0.0539 (15)	0.0015 (12)	0.0063 (13)	−0.0007 (11)
C10	0.091 (2)	0.0568 (17)	0.0607 (17)	0.0001 (16)	0.0024 (16)	0.0107 (14)
C11	0.077 (2)	0.0480 (16)	0.086 (2)	0.0088 (14)	−0.0138 (16)	0.0051 (15)
C12	0.0624 (17)	0.0590 (18)	0.080 (2)	0.0144 (14)	−0.0061 (15)	−0.0181 (16)
C13	0.0539 (15)	0.0590 (17)	0.0530 (15)	0.0080 (13)	−0.0020 (12)	−0.0096 (12)

### Geometric parameters (Å, º)

**Table:**

Br1—C1	1.900 (3)	C5—C7	1.455 (4)
N1—C8	1.383 (3)	C7—H7	0.9300
N1—H1	0.8600	C8—C13	1.389 (4)
N2—C7	1.282 (3)	C8—C9	1.390 (4)
N2—N1	1.344 (3)	C9—C10	1.376 (4)
N3—C1	1.317 (3)	C9—H9	0.9300
N3—C5	1.345 (3)	C10—C11	1.369 (4)
C1—C2	1.377 (4)	C10—H10	0.9300
C2—C3	1.381 (4)	C11—C12	1.375 (5)
C2—H2	0.9300	C11—H11	0.9300
C3—C4	1.366 (4)	C12—C13	1.375 (4)
C3—H3	0.9300	C12—H12	0.9300
C4—C5	1.398 (4)	C13—H13	0.9300
C4—H4	0.9300		
			
C7—N2—N1	117.7 (2)	N2—C7—H7	120.2
N2—N1—C8	120.5 (2)	C5—C7—H7	120.2
N2—N1—H1	119.7	N1—C8—C13	118.9 (2)
C8—N1—H1	119.7	N1—C8—C9	122.4 (2)
C1—N3—C5	117.0 (2)	C13—C8—C9	118.7 (3)
N3—C1—C2	125.9 (3)	C10—C9—C8	119.7 (3)
N3—C1—Br1	116.18 (19)	C10—C9—H9	120.2
C2—C1—Br1	117.9 (2)	C8—C9—H9	120.2
C1—C2—C3	116.3 (3)	C11—C10—C9	121.8 (3)
C1—C2—H2	121.8	C11—C10—H10	119.1
C3—C2—H2	121.8	C9—C10—H10	119.1
C4—C3—C2	120.0 (3)	C10—C11—C12	118.5 (3)
C4—C3—H3	120.0	C10—C11—H11	120.7
C2—C3—H3	120.0	C12—C11—H11	120.7
C3—C4—C5	119.1 (3)	C13—C12—C11	121.0 (3)
C3—C4—H4	120.4	C13—C12—H12	119.5
C5—C4—H4	120.4	C11—C12—H12	119.5
N3—C5—C4	121.5 (2)	C12—C13—C8	120.4 (3)
N3—C5—C7	115.9 (2)	C12—C13—H13	119.8
C4—C5—C7	122.5 (2)	C8—C13—H13	119.8
N2—C7—C5	119.7 (2)		
			
C7—N2—N1—C8	179.8 (2)	N3—C5—C7—N2	−179.5 (2)
C5—N3—C1—C2	−0.6 (4)	C4—C5—C7—N2	0.2 (4)
C5—N3—C1—Br1	178.54 (17)	N2—N1—C8—C13	−178.5 (2)
N3—C1—C2—C3	0.3 (4)	N2—N1—C8—C9	0.3 (4)
Br1—C1—C2—C3	−178.8 (2)	N1—C8—C9—C10	−177.6 (3)
C1—C2—C3—C4	0.2 (5)	C13—C8—C9—C10	1.2 (4)
C2—C3—C4—C5	−0.3 (5)	C8—C9—C10—C11	−0.9 (5)
C1—N3—C5—C4	0.4 (4)	C9—C10—C11—C12	0.2 (5)
C1—N3—C5—C7	−179.9 (2)	C10—C11—C12—C13	0.2 (5)
C3—C4—C5—N3	0.0 (4)	C11—C12—C13—C8	0.1 (5)
C3—C4—C5—C7	−179.6 (3)	N1—C8—C13—C12	178.0 (3)
N1—N2—C7—C5	179.0 (2)	C9—C8—C13—C12	−0.8 (4)

### Hydrogen-bond geometry (Å, º)

**Table:**

*D*—H···*A*	*D*—H	H···*A*	*D*···*A*	*D*—H···*A*
N1—H1···N3^i^	0.86	2.34	3.180 (3)	166

Symmetry code: (i) −*x*+2, *y*−1/2, −*z*+1/2.
